# Loading of AgNPs onto the surface of boron nitride nanosheets for determination of scopoletin in *Atractylodes macrocephala*

**DOI:** 10.1038/s41598-019-40511-y

**Published:** 2019-03-07

**Authors:** Yinzi Yue, Li Zeng, Xiaopeng Wang, Lianlin Su, Mingming Sun, Bensheng Wu, Shuai Yan

**Affiliations:** 1 Department of General Surgery, Suzhou Hospital of Traditional Chinese Medicine, 18 Yangsu Road, 215009 Suzhou, China; 2 The First Clinical Medical College of Nanjing University of Chinese Medicine, 138 Xianlin Avenue, 210023 Nanjing, China; 3 Department of Anorectal Surgery, Suzhou Hospital of Traditional Chinese Medicine, 18 Yangsu Road, 215009 Suzhou, China; 4 School of Pharmacy, Nanjing University of Chinese Medicine, 138 Xianlin Avenue, 210023 Nanjing, China

## Abstract

In this work, silver nanoparticles prepared by a molten salt method were deposited onto the surface of hexagonal boron nitride nanosheet (NS/AgNP) to from a composite. The synthesized nanocomposite was applied for surface modification of screen-printed electrode (SPE). The modified electrode showed a superior performance for electrochemical detection of scopoletin. The electrochemical behaviour of NS/AgNP/SPE was studied in detail. An electrocatalytic oxidation was observed and used for analytical determination of scopoletin concentration. The response of the proposed electrochemical sensing platform was linear over a wide detection range of 2 μM to 0.45 mM with a low limit of detection (LOD) of 0.89 μM. The NS/AgNP/SPE also showed excellent reproducibility and anti-interference property. In addition, the proposed scopoletin sensor was successfully used for the determination of scopoletin in *Atractylodes macrocephala* herb samples.

## Introduction

Hexagonal boron nitride (h-BN) nanosheets, also known as “white graphene”, have a layered structure of hexagonal boron nitride planes^1,2^. They have unique properties that are different from graphene, including a wide bandwidth, insulative characteristics, UV photoluminescence, high thermal conductivity and stability, oxidation resistance and chemical inertness^3–9^. In addition, the polarity of the nitrogen-boron bond and the high surface area of the h-BN nanosheets enable them to adsorb a variety of substances from aqueous solutions. Currently, the preparation of h-BN nanosheets mainly includes solid-state reactions, solvothermal methods, self-propagating high-temperature synthesis and chemical vapour deposition. However, organic solvents are used as the raw materials, the reaction cycles are long, the reaction processes of SHS are difficult to control, and CVD methods have high costs and low yields, which makes it difficult to meet the requirements of industrial production^10–13^.

The molten salt method is a simple and reproducible method for the preparation of inorganic powder materials^14–17^. This method uses safe, non-toxic and recyclable molten salts as a medium to promote the diffusion of the reactants. It has the advantages of low synthesis temperatures, short reaction times, high purities and small particle sizes. In recent years, molten salt methods have been widely used to synthesize various nitrides, such as TiN^18^ and Si_3_N_4_^19^. Scholars, such as Ye^20^, have studied the preparation of h-BN powders by a molten salt method using borax as the boron source, magnesium metal powder as the reducing agent, and chlorine. Thus, h-BN nanosheet powders were synthesized by reaction of potassium carbide in a molten salt medium at 800–1300 °C for 3 h. However, this reaction method requires the use of magnesium powder as a reductant. Moreover, the reaction temperature was high (above 1200 °C), and the product needed to be acid washed. Alternatively, h-BN nanosheets have been prepared by a molten salt nitriding method using NaCl-KCl as the molten salt medium and borax and melamine as the raw materials.

Silver nanoparticles (AgNPs) have good electrocatalytic activities, but their dispersibilities are poor. AgNPs are prone to agglomeration in solvents, making it difficult for them to be used in practical applications^21,22^. To avoid this drawback, AgNPs are often loaded onto the surface of other materials to study their catalytic activity. In this study, h-BN nanosheets were first prepared as the substrate, and sodium citrate was used as a reducing agent to prepare h-BN nanosheet/silver nanoparticle (NS/AgNP) composites. The prepared NS/AgNP composite was used to produce surface-modified screen-printed electrodes (SPEs), which were subsequently applied for electrochemical sensing. The synthetic method for this material was simple, the raw materials were inexpensive and easy to obtain, and the preparation conditions were mild.

Scopoletin is widely found in the roots of *Atractylodes macrocephala*. Studies have shown that scopoletin has pharmacological properties, such as analgesic, anti-inflammatory, antihypertensive and spasmolytic effects, especially in anti-tumour and hyperuricaemia treatments. Scopoletin has attracted wide attention both in China and abroad. To date, several methods have been developed for the determination of scopoletin, including fluorometry^23^, liquid chromatography^24^, spectrofluorimetry^25^ and HPLC-UV^26^ methods. Although these methods have good sensitivities and selectivities, most of them require time-consuming and complex sample pretreatment processes and expensive, large-scale equipment. Electrochemical sensing has the characteristics of rapid analysis, high sensitivity, and simple and portable instrumentation; thus, the study of convenient, fast and sensitive modified electrode materials for detection applications has attracted increasing attention. In this work, we demonstrate the first use of the prepared NS/AgNP composite for the electrochemical sensing of scopoletin. After optimization, a NS/AgNP-modified screen-printed electrode could linearly detect scopoletin over the concentration range of 2 to 450 μM with a lower detection limit of 0.89 μM. In addition, the proposed approach was successfully used to determine the scopoletin content in an *Atractylodes macrocephala* extract.

## Experiments

### Materials

*Atractylodes macrocephala* roots were purchased from a local supermarket in Suzhou, China. Borax was purchased from the Sinopharm Chemical Reagent Co., Ltd. Melamine, sodium citrate and silver nitrite were purchased from Tianjin Tyrande Chemical Reagent Co., Ltd. All other chemicals were analytical grade reagents and were used without further purification.

### Preparation of the h-BN nanosheets

The h-BN nanosheets were prepared using NaCl and KCl as the molten salt medium and melamine and borax as the reactants. The mass ratio of NaCl to KCl was 44:56. The mass ratios of nitrogen to boron were 1:1, 2:1, and 4:1. The components were mixed evenly and put into an alumina crucible. The mixture was dried at 80 °C for 5 h. The mixture was then reacted for 3 h in a GSL-1600X corundum tube furnace at 900–1200 °C under a circulating nitrogen atmosphere. The product was washed and filtered with deionized water and then dried.

### Preparation of the NS/AgNP nanocomposite

The h-BN nanosheets (30 mg) and AgNO_3_ (30 mg) were dispersed into 100 mL and 50 mL of water for 20 min, respectively. After mixing the two dispersions, 30 mL of a sodium citrate solution (3%) was added quickly and stirred for 20 min. After the reaction was completed, the upper layer of the solution was left in static stratification, and the remaining portion was filtered and cleaned repeatedly with ethanol and deionized water. The NS/AgNP composite was then obtained by vacuum drying at 50 °C.

### Characterization

The morphology of the as-synthesized AuNPs was observed using field emission scanning electron microscopy (FESEM) on a ZEISS SUPRA 55. The crystal phase of the sample was characterized by XRD over the 2θ range of 6° to 80° with Cu Kα (*λ* = 0.1546 nm) radiation (D8-Advanced, Bruker).

### Electrode modification and electrochemical measurements

For the electrochemical investigation, a screen-printed electrode (SPE) was rinsed with water. For the electrode surface modification, 5 μL of a catalyst dispersion (0.5 mg/mL) was dropwise added onto the SPE surface and dried at room temperature. Electrochemical measurements were performed on a CH Instruments 660 A electrochemical workstation (CHI-660 A, CH Instruments, Texas, USA). The following conditions were used for the chronocoulometric analysis: initial E(V) = 0.5, final E (V) = 0.25, pulse width (s) = 0.25, step size = 1, and sample time (s) = 2.5 × 10^−4^.

## Results and Discussion

**Figure 1:**
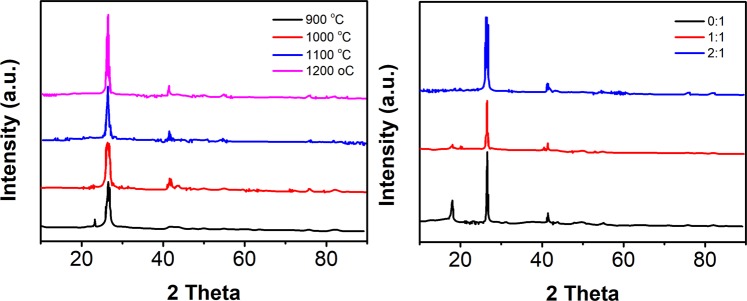
(**A**) XRD patterns of the h-BN nanosheets formed at different temperatures with a salt-to-reactant ratio of 2:1 and a N-to-B ratio of 2:1. (**B**) XRD patterns of the h-BN nanosheets formed at 1000 °C with a N-to-B ratio of 2:1 and different ratios of salt to reactant.

The effects of various h-BN nanosheet preparation parameters were studied. Figure 1A shows the effect of the reaction temperature on the phase composition of the product. When the reaction temperature was 900 °C or 1000 °C degrees, the product was mainly composed of the h-BN phase with a small amount of impurities. When the reaction temperature was increased to 1100 °C and 1200 °C, the product was in the h-BN phase, and no phase impurities could be detected. Compared to the traditional borax-melamine solid-state reaction method, the reaction temperature necessary for obtaining the pure h-BN phase by the molten salt nitriding method was decreased by approximately 100 °C, which is in good agreement with the results of a previous report^20^. The effects of the salt-to-reactant ratio on the phase composition of the products are shown in Fig. 1B. When the ratio of nitrogen to boron was 2:1 and the reaction temperature was 1000 °C, the h-BN phase was present in the products of the reactions with different salt-to-reactant ratios. When the ratio of salt to reactants was increased from 0:1 to 1:1, the diffraction peaks of the phase impurities were still present in the reaction products, but the intensities of the impurity diffraction peaks decreased, indicating that the purity of the h-BN phase increased. When the ratio of salt to reactants was increased to 2:1, the diffraction peaks of the phase impurities disappeared, and only the h-BN phase was observed in the product, which indicates that an increase in the molten salt content promotes the formation of h-BN^27^. This phenomenon happens because complete dissolution of the reactants into the molten salts is conducive to their diffusion and interaction, thus promoting the formation of h-BN. Therefore, the h-BN nanosheets synthesized using a 2:1 ratio of salt to material, a 2:1 ratio of N to B and a temperature of 1100 °C were used for the composite synthesis.

**Figure 2:**
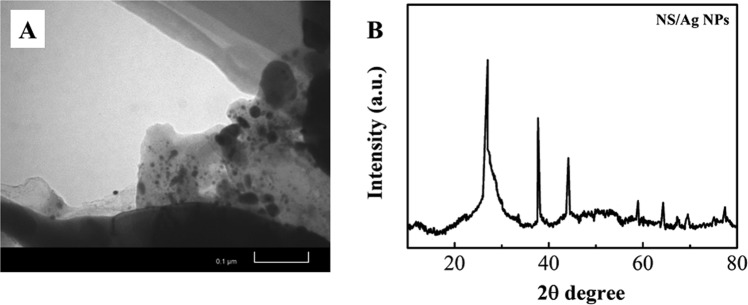
(**A**) TEM image and (**B**) XRD pattern of the NS/AgNPs.

Figure 2A shows a TEM image of the synthesized NS/AgNP composite. The AgNPs can be clearly seen on the h-BN nanosheet surface. The XRD pattern of the NS/AgNP composite is shown in Fig. 2B. The characteristic peaks at 38.3°, 44.8°, 64.2° and 77.4° in the XRD pattern correspond to the (111), (200), (220) and (331) crystal faces of face-centred cubic (fcc) Ag.

**Figure 3:**
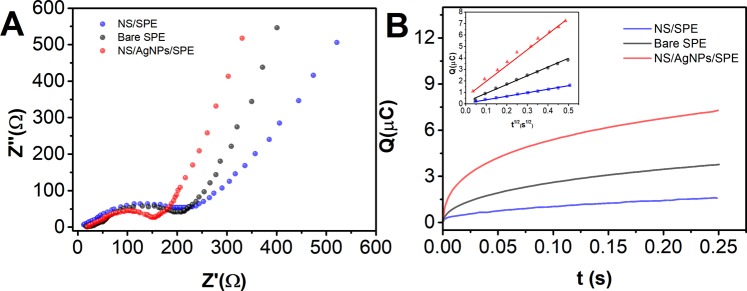
(**A**) Electrochemical impedance spectra and (**B**) Q-t curves of the bare SPE, NS/SPE and NS/AgNP/SPE in a 0.1 M KCl electrolyte with 5 mM [Fe(CN)_6_]^3−/4−^.

Electrochemical impedance spectroscopy (EIS) was used to investigate the electrochemical properties of a bare SPE, an NS/SPE and an NS/AgNP/SPE using 5 mM [Fe(CN)_6_]^3−/4−^ in 0.1 M KCl as the electrolyte. As shown in Fig. 3A, the EIS spectrum of the NS/SPE shows a slightly larger semicircle than that of the bare SPE due to the insulating nature of the h-BN nanosheets. In contrast, the NS/AgNP/SPE shows a much smaller semicircle, indicating that the surface modification improves the electron transfer rate on the SPE surface.

We further used 0.1 mM K_3_[Fe(CN)_6_] as a probe molecule for calculating the effective electrode area of the different modified electrodes using chronocoulometry (Fig. 3B). According to the Anson equation^28^:

$$Q=\frac{2nFAc{D}^{\frac{1}{2}}}{{n}^{\frac{1}{2}}}{t}^{1/2}+{Q}_{dl}+{Q}_{ads}$$

where c is the substrate concentration, D is the diffusion coefficient (in 0.1 M KCl solution, the diffusion coefficient for 0.1 mM K_3_[Fe(CN)_6_] is 7.6 × 10^−6^ cm^2^/s), n is the electron transfer number, Q_dl_ is the double-layer charge (which can be eliminated by subtracting the background signal), Q_ads_ is the faradaic charge, A is the apparent surface area of the SPE, and F is the Faraday constant (F = 96485 C/M). The effective area, A, values of the bare SPE, NS/SPE and NS/AgNP/SPE were 0.10, 0.25 and 0.4 cm^2^, respectively. The results show that the h-BN nanosheets modified with AgNPs had a larger specific surface area and a greater enrichment effect toward the target molecule than those of the other electrodes.

**Figure 4:**
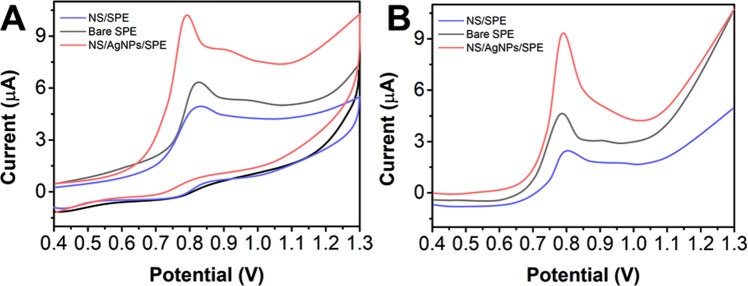
(**A**) Cyclic voltammograms and (**B**) differential pulse voltammetry (DPV) curves of the bare SPE, NS/SPE and NS/AgNP/SPE towards a 50 μM solution of scopoletin.

The CV and DPV curves of scopoletin with the different modified electrodes in a PBS solution at pH 7 were compared to study the effects of the composite on the electrocatalytic activity. Figure 4A shows the CV comparison of a 50 μM scopoletin solution obtained with the different modified electrodes. The oxidation peak current of scopoletin increases gradually from the bare SPE to the NS/SPE and to the NS/AgNP/SPE, indicating that the electrochemical activity toward scopoletin is enhanced by the presence of the silver nanoparticles and h-BN nanosheets. A comparison of the SWV data (Fig. 4B) shows that the NS/AgNP/SPE exhibits a superior electrocatalytic activity toward scopoletin and that its peak current was significantly greater than those of the other electrodes, which improved the detection sensitivity.

**Figure 5:**
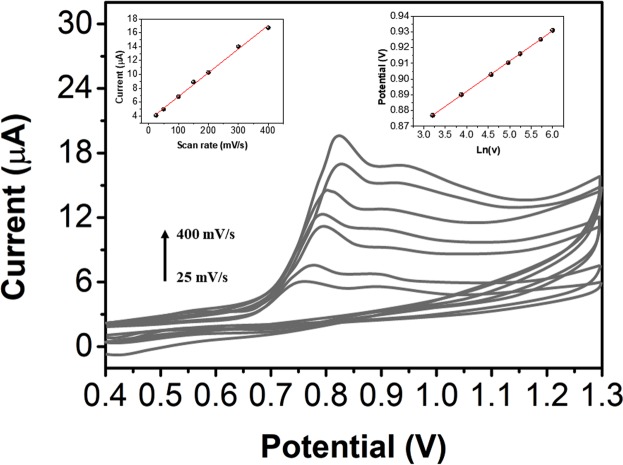
CV curves of a 50 μM solution of scopoletin on the NS/AgNP/SPE in a pH 7 PBS solution at different scan rates.

The CV curves of 50 a μM scopoletin solution obtained with the NS/AgNP/SPE at different scanning rates are shown in Fig. 5. The peak current (I_p_) of scopoletin increased linearly with increasing scanning rates (v) from 25 mV/s to 400 mV/s and followed the equation I_p_ = 3.3418 + 3.42006 v (R^2^ = 0.996), indicating that the oxidation of scopoletin at the NS/AgNP/SPE surface was an adsorption-controlled process. The effect of the scanning rate on E_p_ is shown in the inset of Fig. 5. Both E_p_ and v conform to the following equation:

$${E}_{P}=K+\frac{RT}{{\rm{an}}F}\,\mathrm{ln}\,v$$

Because the process at the electrode surface is controlled by adsorption, the relationship between E_p_ and v follows the equation:

$${E}_{P}={E}^{0}+(\frac{RT}{anF})\mathrm{ln}(\frac{RT{K}^{0}}{anF})+\frac{RT}{anF}\,\mathrm{ln}\,v$$

where K^0^ is the standard rate constant of the surface reaction, E^0^ is the standard electrode potential, and α is the transfer coefficient for scopoletin oxidation. E_p_ shows a linear relationship with v, and the value of α can be obtained from the slope, which is 0.021. Given that α = 0.5 is obtained, the n value of scopoletin is 2. Therefore, the oxidation process of scopoletin involves two electrons.

**Figure 6:**
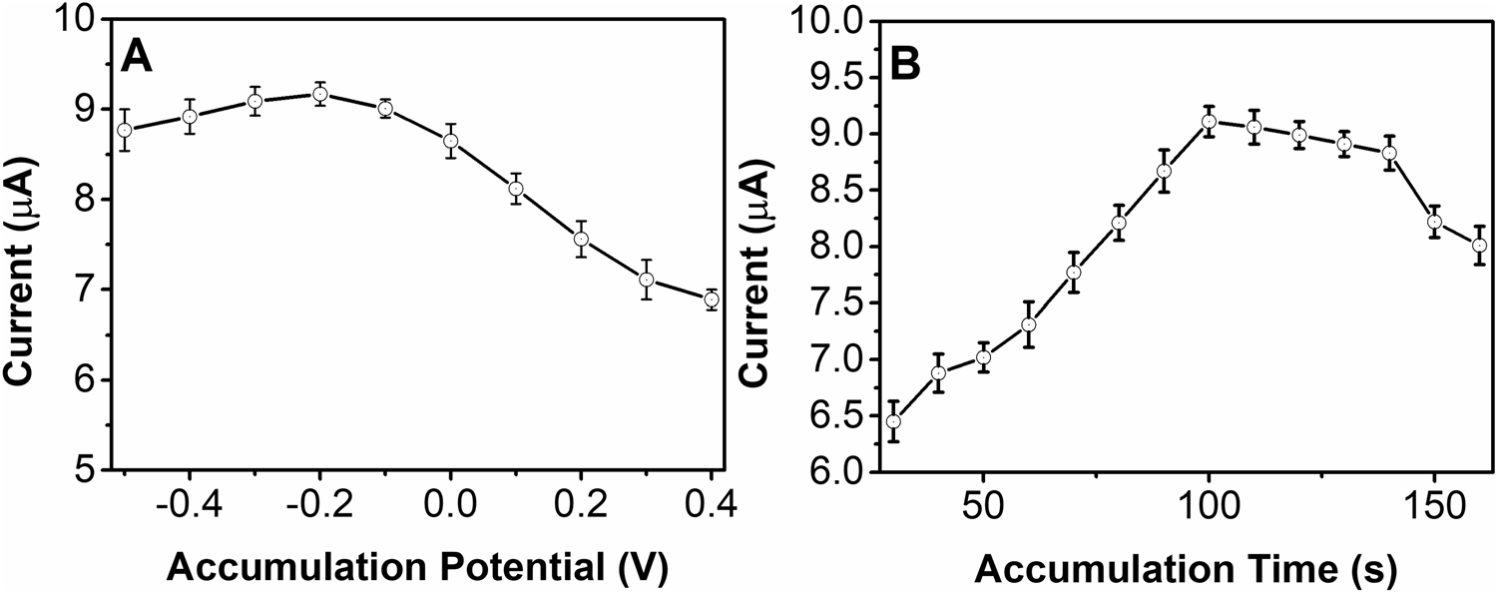
Effects of (**A**) accumulation potential and (**B**) accumulation time of the NS/AgNP/SPE towards a 50 μM solution of scopoletin.

Accumulation is a very useful pretreatment process in electrochemical sensing. Figure 6A shows the effects of the oxidation of a 50 μM solution of scopoletin at the NS/AgNP/SPE with different accumulation potentials. The results suggests that the oxidation response increases when the accumulation potential decreases from 0.4 to −0.2 V. The maximum performance was observed at an accumulated potential of −0.2 V. Additionally, further lowering the accumulation potential decreased the sensing performance. Therefore, −0.2 V was selected as the optimal accumulation potential for scopoletin oxidation. The accumulation time is another crucial parameter during accumulation pretreatment. As shown in Fig. 6B, the current response of scopoletin oxidation increases with increasing accumulation time. A significant current increase can be observed over the accumulation times of 30 to 100 s. The enhancement rate of the current response decreases after 100 s, especially after 140 s. Therefore, 100 s was selected as the accumulation time.

**Figure 7:**
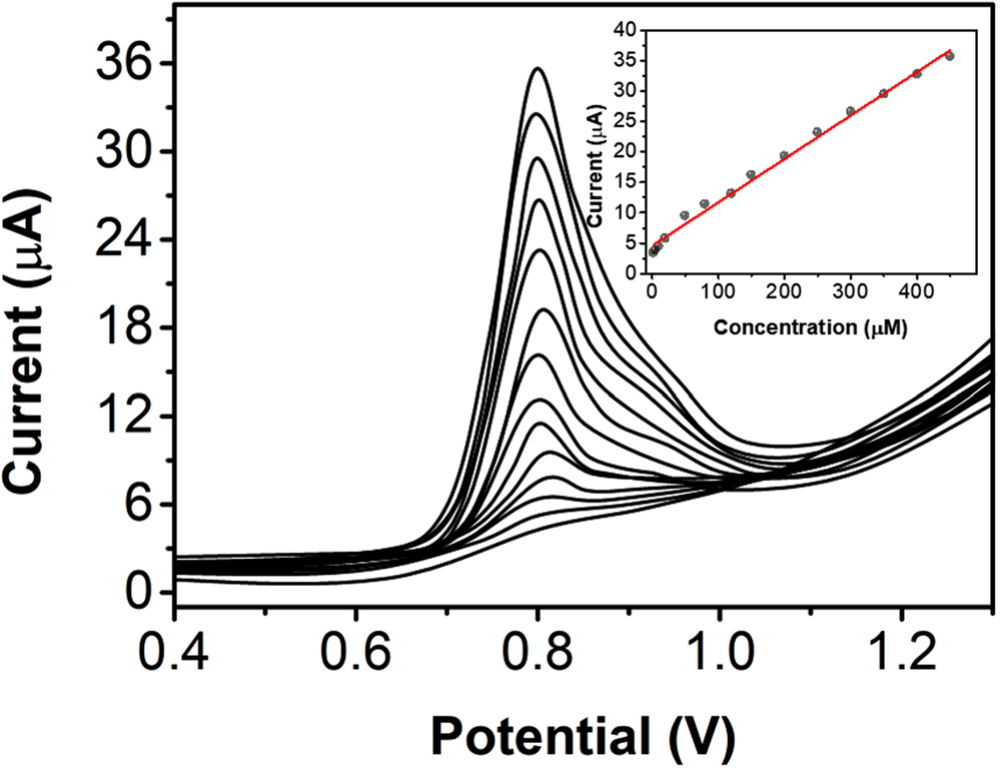
Differential pulse voltammograms of the NS/AgNP/SPE towards scopoletin at concentrations ranging from 50 nM to 0.2 mM.

The analytical sensing ability of the NS/AgNP/SPE towards scopoletin was investigated using differential pulse voltammetry (DPV) due to the high sensitivity of this method. Figure 7 shows the DPV curves of the NS/AgNP/SPE towards scopoletin at concentrations ranging from 2 μM to 0.45 mM. The inset shows the plots of the current response against the scopoletin concentration. A linear response was observed and can be expressed as I(μA) = 0.07144 C + 4.57122 (R^2^ = 0.993). The lower limit of detection was calculated to be 0.89 μM based on a signal-to-noise ratio of 3. Based on the above results, the NS/AgNP/SPE exhibits excellent electrochemical sensing capabilities and can be used for scopoletin detection in herb samples. Table 1 summarizes the detection performance of our work compared with those of previous reports.

**Table 1:** Analytical performance comparison of scopoletin determination methods.

Analytical method	Detection range	Limit of detection	Reference
LC–MS/MS	26 nM to 536 nM	—	^29^
HPLC-UV	0.86 μM to 31.51 μM	—	^26^
Fluorescence Spectrometry	0.31 μM to 2.2377 μM	—	^30^
RP-HPLC	26.02 nM to 156.12 nM	2.03 nM	^31^
Electrochemical sensor	2 μM to 0.45 mM	0.89 μM	This work

Dry *Atractylodes macrocephala* was used as a real sample for investigating the practical performance of the NS/AgNP/SPE towards scopoletin detection. A standard addition process was used, and the results are shown in Table 2. The results show that the average scopoletin concentration in *Atractylodes macrocephala* was 5.478 μM. HPLC (Agilent 1100) was used to confirm the electrochemical sensing performance.

**Table 2:** Electrochemical detection of scopoletin in *Atractylodes macrocephala* and the recovery results.

Sample	Amount detected (μM)	HPLC (μM)	Added (μM)	Amount detected (μM)	Recovery (%)	RSD (%)
1	6.441	6.316	5	11.420	99.82	2.78
2	5.251	5.250	5	10.378	98.83	2.61
3	5.239	5.235	10	14.725	96.63	3.19
4	4.980	4.782	10	15.107	100.85	4.02

**Figure 8:**
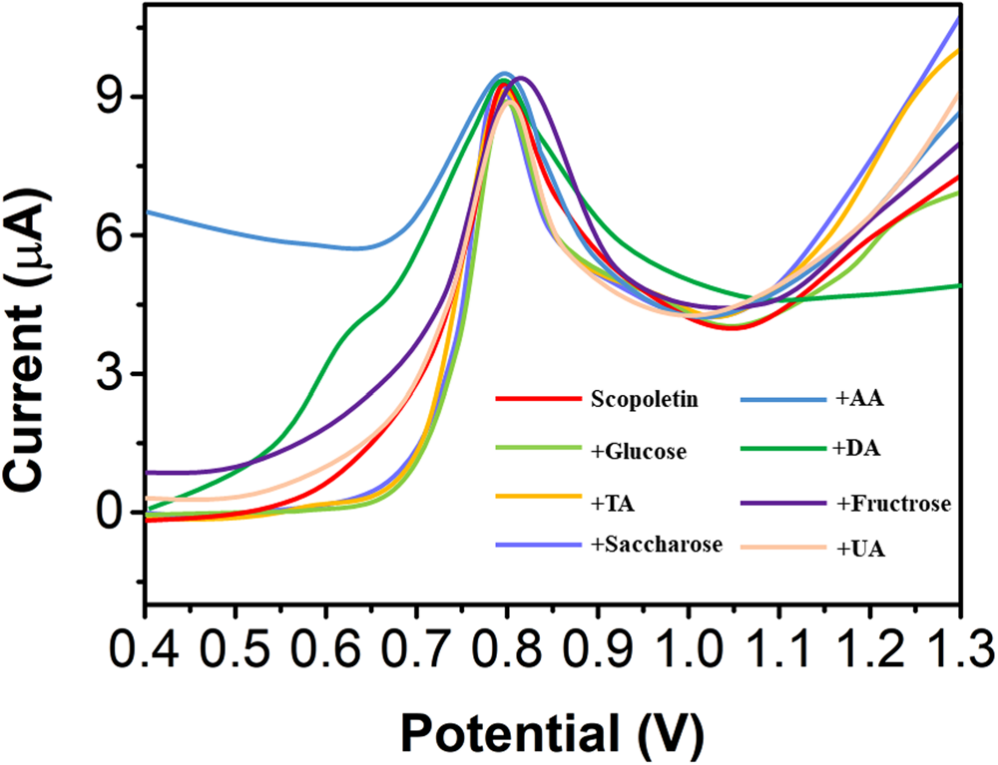
I-T response of the NS/AgNP/SPE with successive additions of 50 μM of scopoletin, glucose, tartaric acid, uric acid, ascorbic acid, dopamine, saccharose and fructose.

The anti-interference properties of the NS/AgNP/SPE were also investigated. An I-T experiment was conducted with the successive additions of 50 μM scopoletin, glucose, tartaric acid (TA), uric acid (UA), ascorbic acid (AA), dopamine (DA), saccharose and fructose (Fig. 8). All interference species showed negligible effects on scopoletin detection (current changes of less than 5%). The results indicate that the proposed NS/AgNP/SPE has excellent anti-interference properties for scopoletin detection.

**Figure 9:**
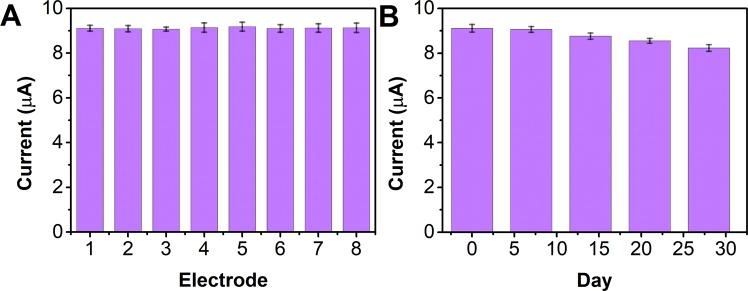
(**A**) Reproducibility of eight individual NS/AgNP/SPEs detecting a 50 μM solution of scopoletin. (**B**) Long-term stability test of the NS/AgNP/SPE.

The reproducibility of the NS/AgNP/SPE was investigated with eight individually prepared NS/AgNP/SPEs. Figure 9A shows the current responses of the eight NS/AgNP/SPEs towards a 50 μM solution of scopoletin. The RSD for the electrodes was calculated to be 5.11%, suggesting that the proposed electrode has a stable performance. The long-term stability of the electrodes was also tested. As shown in Fig. 9B, the electrode retained more than 90% of its original performance after 1 month of storage at room temperature, suggesting its acceptable long-term stability.

## Conclusions

In conclusion, h-BN nanosheets were first prepared using a molten salt method using borax and melamine as the raw materials. Then, the NS/AgNP composite was prepared by a mild citric acid-assisted reduction process. The prepared NS/AgNPs were used for SPE surface medication and subsequently applied for the electrochemical determination of scopoletin. Under optimal conditions, the NS/AgNPs/SPE showed a linear detection capability towards scopoletin over a concentration range of 2 μM to 0.45 mM with a lower detection limit of 0.89 μM. In addition, the proposed NS/AgNPs/SPE was used for the determination of scopoletin in dry *Atractylodes macrocephala* samples.
